# Down-regulation of the Lamin A/C in neuroblastoma triggers the expansion of tumor initiating cells

**DOI:** 10.18632/oncotarget.5104

**Published:** 2015-09-03

**Authors:** Marta Nardella, Loredana Guglielmi, Carla Musa, Ilaria Iannetti, Giovanna Maresca, Donatella Amendola, Manuela Porru, Elisabetta Carico, Giuseppe Sessa, Rosalba Camerlingo, Carlo Dominici, Francesca Megiorni, Marika Milan, Claudia Bearzi, Roberto Rizzi, Giuseppe Pirozzi, Carlo Leonetti, Barbara Bucci, Delio Mercanti, Armando Felsani, Igea D'Agnano

**Affiliations:** ^1^ Institute of Cell Biology and Neurobiology-CNR, Monterotondo, Rome, Italy; ^2^ S.Pietro Hospital Fatebenefratelli, Rome, Italy; ^3^ Regina Elena Cancer Institute, Rome, Italy; ^4^ UOD Cytopathology, Department of Molecular and Clinical Medicine, Faculty of Medicine and Psychology, Sapienza University, Rome, Italy; ^5^ Department of Experimental Oncology, National Cancer Institute –IRCCS “Fondazione G. Pascale”, Naples, Italy; ^6^ Department of Paediatrics and Infantile Neuropsychiatry, Sapienza University, Rome, Italy; ^7^ School of Reproductive and Developmental Medicine, University of Liverpool, Liverpool, United Kingdom; ^8^ I.R.C.C.S Multimedica, Scientific and Technology Pole, Milan, Italy

**Keywords:** neuroblastoma, TICs, Lamin A/C, MYCN, miRNAs

## Abstract

Tumor-initiating cells constitute a population within a tumor mass that shares properties with normal stem cells and is considered responsible for therapy failure in many cancers. We have previously demonstrated that knockdown of the nuclear envelope component Lamin A/C in human neuroblastoma cells inhibits retinoic acid-mediated differentiation and results in a more aggressive phenotype. In addition, Lamin A/C is often lost in advanced tumors and changes in the nuclear envelope composition occur during tumor progression. Based on our previous data and considering that Lamin A/C is expressed in differentiated tissues, we hypothesize that the lack of Lamin A/C could predispose cells toward a stem-like phenotype, thus influencing the development of tumor-initiating cells in neuroblastoma. This paper demonstrates that knockdown of Lamin A/C triggers the development of a tumor-initiating cell population with self-renewing features in human neuroblastoma cells. We also demonstrates that the development of TICs is due to an increased expression of MYCN gene and that in neuroblastoma exists an inverse relationship between LMNA and MYCN expression.

## INTRODUCTION

Neuroblastoma (NB) is the most common childhood (15% of deaths in children) extracranial tumor of the autonomic nervous system that originates from neural crest-derived sympathoadrenal progenitors. The clinical behavior of NB is correlated with specific genomic abnormalities and high level amplification of the MYCN oncogene [1]. Treatment for patients with advanced disease is aggressive multimodal therapy (e.g. intensive chemotherapy, radiation, surgery, hematopoietic stem cell rescue and immunotherapy) even though the survival of these patients remains at 40–50%.

A reservoir of tumor cells (within a tumor) that has similar properties to normal stem cells might be responsible for therapy failure during long-term remission. This subpopulation of cells has been termed cancer stem cells (CSCs) or tumor-initiating cells (TICs). Although hematopoiesis is the most thoroughly studied stem cell system, local resident stem cells could maintain several other organs and many solid tumors, including NB, may contain a population of TICs [2, 3]. However, we know little about the phenotype of NB-TICs. To individuate TICs within a tumor, researchers normally focus on the study of functional stem cell-like characteristics such as self-renewal, tumorigenicity and drug resistance [4].

In a previous paper, we have shown that knockdown of Lamin A/C expression in NB cells inhibits cell differentiation and gives rise to a more aggressive and drug-resistant tumor phenotype [5]. Lamins, which are type V intermediate filaments, are the major components of the nuclear lamina. They are divided into A and B types based on similarities in their primary sequences and biological properties; A- and B-type lamins are expressed in differentiated tissues and ubiquitously, respectively [6, 7]. Additionally, the expression of A-type lamins is often reduced or absent in cells that are highly proliferative, including various malignancies [8, 9]. Based on our previous data and considering the different expression of Lamin A/C during development, we hypothesize that the lack of Lamin A/C could predispose cells toward a more immature phenotype. The aim of the present paper is to investigate whether Lamin A/C expression levels could influence the presence of TICs in NB.

Moreover, we seek to study the mechanism by which Lamin A/C could control tumor aggressiveness in NB. Changes in the composition of the nuclear membrane occur during the progression of several types of cancers [10, 11]. Good functionality of the nuclear membrane could play a crucial role in biological functions such as microRNA (miRNA) processing. miRNA expression is a complex process that requires an initial step of maturation in the nucleus, an intermediate phase of nuclear export, and a final processing of the pre-miRNA into the mature miRNA in the cytoplasm [12]. miRNAs are critical in controlling the self-renewal and differentiation of embryonic stem cells [13] and aberrant expression and/or functions of miRNAs are associated with tumorigenesis and the metastasis in many forms of cancer, including NB [14, 15]. The mechanisms leading to miRNA dysregulation in NB are still not fully understood. Several miRNAs, including the miR-17–92 cluster, are induced by the NB-specific transcription factor MYCN [16–18]. On the other hand, certain miRNAs, such as miR-101, influence MYCN transcription [19].

The data herein demonstrate that knockdown of Lamin A/C triggers the development of a TIC population with self-renewal features in human NB cells.

## RESULTS

### Abundance of LMNA in NB tumors is inversely correlated with that of MYCN gene

**Figure 1:**
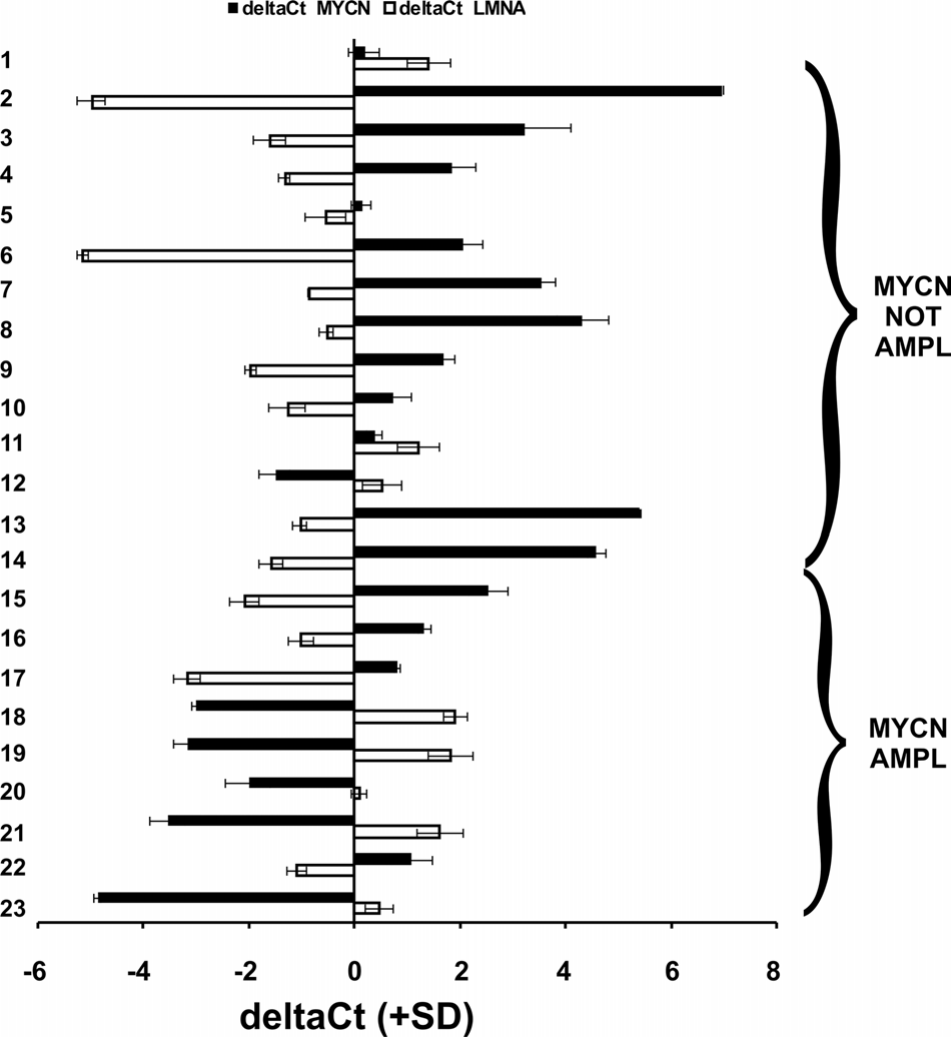
The expression of LMNA and MYCN are inversely correlated in NB human biopsies qRT-PCR analysis of the LMNA (white) and MYCN (black) genes in 23 NB human biopsies. Data (means + SD [*n* = 3]) are reported as the deltaCt values normalized against the endogenous control. The deltaCt values are inversely correlated with the amount of the gene present in the sample. Statistical significance: *p* ≤ 0.01.

We analyzed the expression levels of the LMNA and MYCN genes in 23 NB biopsies obtained from the Department of Pediatrics and Infantile Neuropsychiatry of Sapienza University. The tumors were classified according to the International Neuroblastoma Pathology Classification (INPC) and staged as stages 1 to 4. Nine of 23 cases showed amplification of MYCN DNA. The expression levels of the two genes significantly inversely correlated (*p* = 0.01), independently of the DNA amplification of MYCN, in 21 out of the 23 cases analyzed; i.e., as LMNA increased, MYCN gene expression decreased (Fig. 1).

We decided to study this inverse relationship between LMNA and MYCN gene in an *in vitro* experimental model of NB. We choose the SH-SY5Y and LAN-5 NB cell lines, with N-type morphology. The SH-SY5Y cells express Lamin A/C at high levels and have single copy of MYCN gene [20]; while LAN-5 cells show an amplification of the MYCN gene and do not express Lamin A/C [20]. In particular, since Lamin A/C has been demonstrated to play an epigenetic role in regulating gene expression and miRNAs can be targeted by MYCN, we hypothesized a possible reciprocal regulation between the two genes mediated by miRNAs.

**Figure 2:**
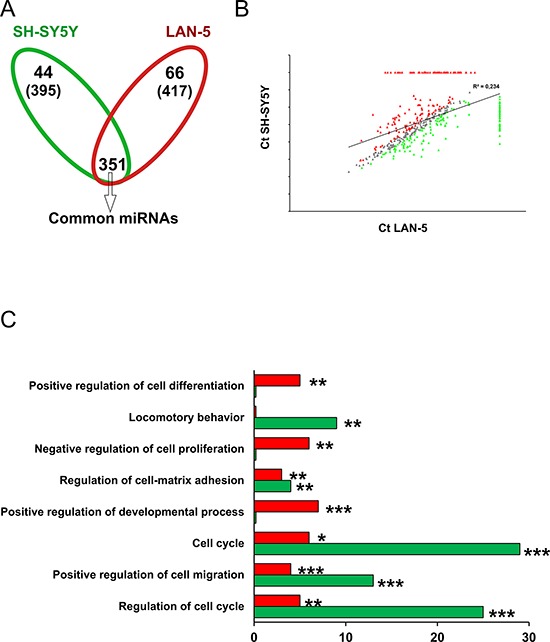
Functional analysis of miRNA target genes in LAN-5 and SH-SY5Y cell lines **A.** Venn diagram of expressed miRNAs in SH-SY5Y and LAN-5 cells. The number in parenthesis represents the total miRNAs expressed in each line. **B.** Scatter plot of the miRNA Ct values normalized against endogenous controls in SH-SY5Y and LAN-5 cells. Grey dots represent unchanged miRNAs between the two cell lines; green dots are the SH-SY5Y miRNAs; red dots represent LAN-5 miRNAs. **C.** Gene ontology by DAVID. In the histogram are reported the number of target genes belonging to the indicated functional categories. Red bar, LAN-5 cells; green bar, SH-SY5Y cells. Statistical significance: *p ≤ 0.05, **p ≤ 0.01, ***p ≤ 0.001.

We performed a miRNA expression profiling of LAN5 and SH-SY5Y cells using TaqMan Human MicroRNA Arrays. A total of 768 miRNAs, present in the array, were analyzed in each cell line. The distribution of the expressed miRNAs is shown in a Venn diagram where a total of 417 (66 specific and 351 common) and of 395 (44 specific and 351 common) miRNAs were found expressed in LAN-5 and SH-SY5Y cells, respectively (Fig. 2A). We found 359 and 337 miRNAs not expressed in SH-SY5Y and LAN-5 cells, respectively (293 not expressed at all in both cell lines). We identified a set of 202 out of the 351 common miRNAs differentially expressed at least 2-fold change between the two cell lines (99 in the LAN-5 and 103 in the SH-SY5Y cells); whereas 149 miRNAs were filtered out by the threshold applied. A scatter plot analysis shows the correlation between miRNA expression values (Ct) in LAN-5 and SH-SY5Y cell lines (Fig. 2B). Grey dots distributed along the bisector line represent miRNAs similarly expressed in the two lines (*n* = 149). While, red or green dots correspond to miRNAs with high expression in the LAN-5 (*n* = 165) and SH-SY5Y (*n* = 147), respectively.

Considering the specifically and the differentially expressed miRNAs we performed a functional analysis using the DIANA-mirPath 2.0 tool, and in particular the software TarBase which uniquely clusters those miRNAs whose targets are experimentally validated [21]. We filtered the clusters obtained based on their significance (FDR corrected *p* < 0.05). As to be expected, target genes resulted grouped into functional categories associated with cancer phenotype. The most modulated miRNAs in both cell lines belong to pathways involved in the regulation of cell proliferation, apoptosis, and response to treatment: “p53 signaling pathway”, “cell cycle”, “pathways in cancer”, “PI3K-Akt signaling pathway”, “transcriptional misregulation in cancer”. These pathways are consistent with the inherent phenotypic characteristics of the two cell lines and correlate to their different capacity to proliferate, to undergo apoptosis, to migrate and invade (Supplementary Table 1). Since a single miRNA can inhibit several target mRNAs and multiple miRNAs can target a single mRNA in a combinatorial fashion, to identify more accurately the differences between the miRNome profiles of these two NB cell lines, we intersected the target genes derived from the two cell lines in order to identify the identical genes which were then removed from the analysis. In Table 1 are reported target genes, and relative miRNAs, identified only in SH-SY5Y or LAN-5 cells after removing the identical genes. The gene ontology performed using the DAVID tool on this subset of target genes evidenced that the most relevant enriched functional categories identified in SH-SY5Y cells were associated with cell migration, locomotion and cell proliferation (Fig. 2C and Table 2). This indicates that SH-SY5Y cells, which express LMNA gene, have a reduced ability to migrate, to proliferate and hence to be aggressive. Whereas, the LAN-5 cells, which instead express MYCN gene, showed an enrichment in functional categories associated with regulation of differentiation and cell proliferation suggesting that these cells are less prone to differentiate and might show a stem-like phenotype with respect to SH-SY5Y cells (Fig. 2C and Table 2). On the basis of these results, we hypothesize that the mutual expression of MYCN and LMNA genes could address NB cells to a stem-like or a differentiating phenotype, respectively.

**Table 1:** Target genes and relative miRNAs specifically expressed only in SH-SY5Y or LAN-5 cells

SH-SY5Y												
KEGGterm	Count	*P* Value	Genes						Count	miRNA|TarBase		
hsa04115:p53 signaling pathway	22	1.08E-29	APAF1	CASP8	SESN1	MDM4	CDKN2A	TP73	18	miR-101-3p	miR-26b-5p	miR-21-5p
			ATM	CCND3	SFN	PIDD1	DDB2	ZMAT3		miR-424-5p	miR-93-5p	miR-193a-5p
			BID	CCNE1	THBS1	RFWD2	EI24	GADD45A		miR-34a-5p	miR-24-3p	miR-374a-5p
			CASP3	CCNG2	TP53I3	SERPINB5						
hsa05200:Pathways in cancer	51	5.47E-24	APPL1	CCNE1	FGF1	ITGA6	MMP2	TP53	36	miR-214-3p	let-7c	miR-222-3p
			AR	CHUK	FGFR1	JUN	MMP9	BMP2		let-7d-5p	miR-21-5p	miR-25-3p
			AXIN2	CRKL	FZD4	LAMC2	NFKBIA	ERBB2		miR-424-5p	miR-363-3p	miR-24-3p
			BID	DAPK1	HDAC1	MAP2K1	NRAS	IKBKB		miR-146b-3p	miR-93-5p	miR-503
			PLD1	STAT1	VHL	RALA	TCF4	MMP1		miR-449a	miR-34a-5p	miR-26a-5p
			PTK2	STAT5A	WNT1	RALB	TFG	PIK3R1		let-7f-5p	miR-223-3p	miR-28-5p
			RB1	TGFB2	RUNX1	TGFBR1	SKP2	PDGFB		miR-27a-3p	miR-29b-3p	miR-374a-5p
			BIRC3	DVL2	HSP90AA1	MAPK1	PDGFA	MAPK9		miR-769-5p	miR-28-5p	miR-361-5p
			BIRC5	E2F3	IGF1R					miR-221-3p	miR-199b-5p	miR-361-5p
hsa04110:Cell cycle	17	6.56E-21	ANAPC13	ATM	CCND3	CCNE1	CDC14A	GADD45A	14	miR-424-5p	miR-34a-5p	let7d-5p
			MDM2	ORC4	SFN	SKP2	TP53	HDAC1		miR-374a-5p	let-7c	miR-25-3p
			CDC23	CDC25C	E2F3	E2F5	EP300			mirR-503	mir-769-5p	
hsa04110:PI3K-Akt signaling pathway	32	1.02E-07	PDGFA	MAP2K2	IGF1R	FGFR1	COL3A1	CASP9	26	let-7c	miR-449a	miR-100-5p
			PDGFB	MDM2	IKBKB	FGFR3	COL4A1	CCNE1		let-7f-5p	miR-503	miR-424-5p
			PDGFC	MYB	INSR	GNG5	COL5A3	CDC37		miR-7-5p	miR-146b-3p	miR-181c-5p
			PIK3CB	NRAS	IRS1	HSP90AA1	EIF4E	CHUK		miR-222-3p	let-7d-5p	miR-29b-3p
			PPP2R1B	PAK1	MAP2K1	IFNB1	FGF1	COL1A1		miR-26a-5p	miR-34b-5p	
			PRKAA1	RAF1								
hsa05202: Transcriptional misregulation in cancer	39	3.14E-03	TGFBR2	PDGFB	MEIS1	Il6	HIST2H3C	HIST1H3H	19	miR-34a-5p	miR-223-3p	miR-363-3p
			TP53	RUNX1	MYC	KLF3	HIST3H3	HIST1H3I		miR-146b-3p	miR-93-5p	miR-25-3p
			ZEB1	SMAD1	MYCN	LMO2	HOXA9	HIST1H3J		miR-27a-3p	miR-26a-5p	miR-101-3p
			SP1	PAX3	MEF2C	IGF1R	HIST2H3A	HIST1H3E		miR-28-5p		
			HIST1H3C	H3F3B	CEBPB	BIRC3	CDKN2C	BAIAP3				
			HDAC1	FOXO1	CCND2	ATM	HIST1H3B	H3F3A				
			HIST1H3A	GOLPH3	CDKN1B							

LAN-5										
KEGGterm	Count	*P* Value	Genes					Count	miRNA|TarBase	
hsa04110:Cell cycle	6	4.05E-32	BUB1B	ORC6	PKMYT1	SMAD3	SMAD2	2	miR-155-5p	miR-193b-3p
			PRKDC							
hsa05200:Pathways in cancer	21	1.12E-08	RAC2	RAD51	TCF7L1	NFKB2	SMAD2	5	miR-155-5p	miR-378a-5p
			CDH1	FN1	SUFU	RHOA	RAC2		miR-193b-3p	miR-200c-3p
			HSP90AB1	CASP9	MAX	NKX3-1	FGF7		miR-504	
			CTNNA1	JUP	SMAD3	FGF2	CDK2			
			BAX							
hsa04115:p53 signaling pathway	2	1.35E-05	BAX	RCHY1				2	miR-504	miR-19a-3p
hsa04110:PI3K-Akt signaling pathway	15	8.07E-03	EIF4E2	FGF2	FGF7	FLT1	GNB4	2		
			ITGB4	ITGB5	RHEB	PKN2	PRKCZ		miR-155-5p	miR-19a-3p
			THBS1	YWHAZ	NFKB1	PCK2	PDK1			
hsa05202:Transcriptional misregulation in cancer	2	4.32E-02	CD40	BCL2L1				2	miR-486-5p	miR-491-5p

**Table 2:** Gene Ontology analysis on the target genes specifically expressed only in SH-SY5Y or LAN-5

SH-SY5Y							
GOTERM	*P* value	Count	Target Genes				
GO:0051726∼regulation of cell cycle	4.84E-16	25	STAT5A	SFN	CCNG2	CDC37	TGFB2
			CASP3	CDKN2A	CDKN2C	INSR	MYC
			BMP2	TP53	SKP2	CDC23	BIRC5
			RB1	CDC25C	ATM	CDKN1B	HDAC1
			CCND3	CCND2	JUN	MDM2	GADD45A
GO:0030335∼positive regulation of cell migration	1.21E-11	13	IL6	PLD1	PDGFB	MAP2K1	PDGFA
			MMP9	IRS1	TGFB2	IGF1R	MAPK1
			THBS1	INSR	PIK3R1		
GO:0007049∼cell cycle	5.21E-11	29	E2F3	CDC14A	ANAPC13	SESN1	CCNG2
			TGFB2	CCNE1	CDKN2A	CDKN2C	THBS1
			MYC,	MAP2K1	TP53	SKP2	CDC23
			BIRC5	RB1,	CDC25C	APPL1	ATM
			TP73	MAPK1	CDKN1B	EP300	CCND3
			CCND2	MDM2	MDM4	GADD45A	
GO:0001952∼regulation of cell-matrix adhesion	0.002	4	CDKN2A	PIK3CB	THBS1	PIK3R1	
GO:0007626∼locomotory behavior	0.003	9	MAPK1	PLD1	IL6	MAP2K1	PDGFB
			PDGFA	PIK3CB	RALA	TGFB2	

LAN-5							
GOTERM	*P* value	Count	Genes				
GO:0051094∼positive regulation of developmental process	9.707E-05	7	BAX	RHOA	SMAD3	NFKB1	SMAD2
			THBS1	FGF2			
GO:0001952∼regulation of cell-matrix adhesion	0.002	3	PRKCZ	SMAD3	THBS1		
GO:0008285∼negative regulation of cell proliferation	0.003	6	BAX	NKX3-1	SMAD3	SMAD2	THBS1
			FGF2				
GO:0045597∼positive regulation of cell differentiation	0.004	5	RHOA	SMAD3	NFKB1	SMAD2	FGF2

**Figure 3:**
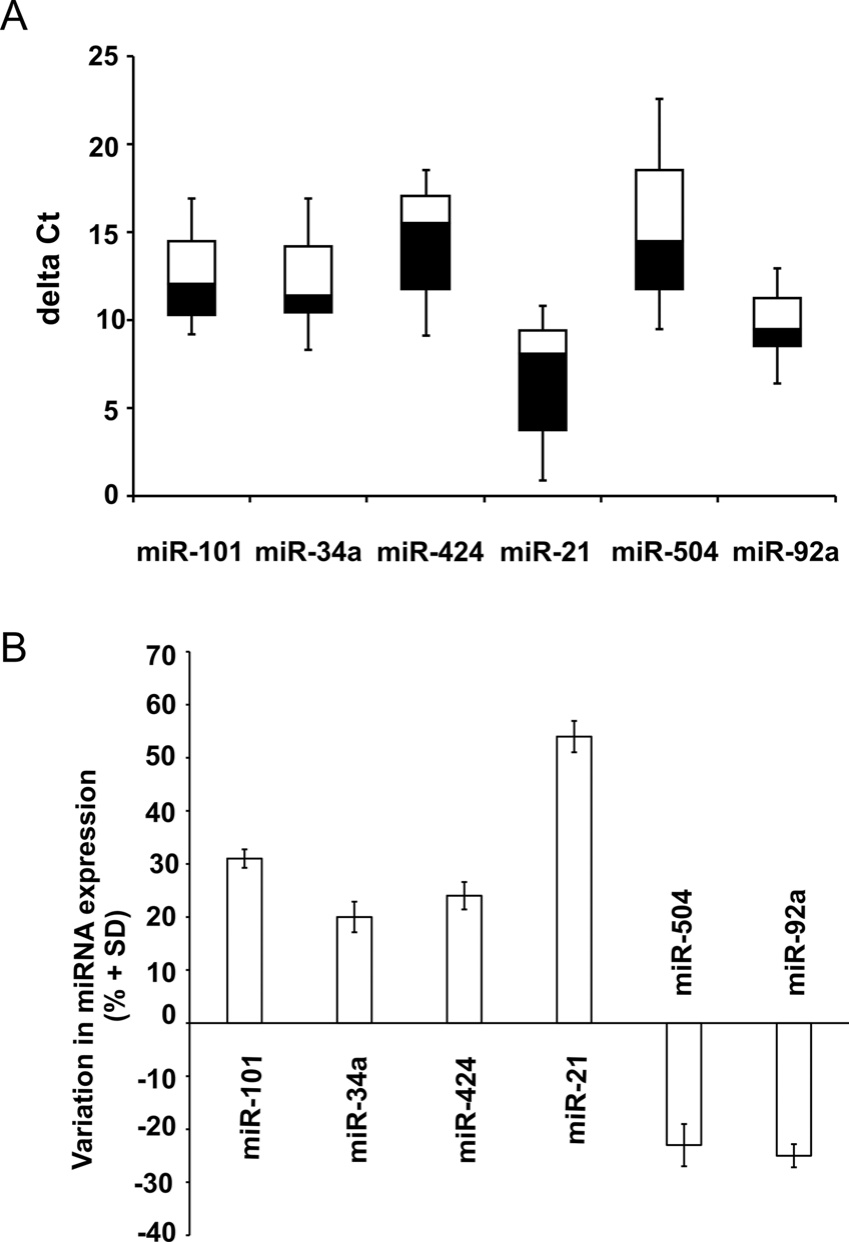
miRNA validation in NB human biopsies **A.** Boxplot diagrams of deltaCt values of the indicated miRNAs. Each box represents the distribution of the deltaCt measured for a single miRNA in 15 tumor tissue specimens. Bottoms and tops of the boxes define the 25^th^ and 75^th^ percentile, the band inside the box defines the median, and the ends of the whiskers define the lowest and the highest value of all data. **B.** Data are expressed as percentage variation (+SD) of the indicated miRNAs in the NB patient group with high LMNA expression compared with the specimen group with low LMNA expression, defining high and low LMNA expression on the LMNA deltaCt median values (threshold = −1.00) (*p* = 0.008).

To validate the results obtained in cell cultures we analyzed the expression of some selected miRNAs in tumor tissue RNA specimens previously studied for LMNA and MYCN expression. Among the most differentially expressed miRNAs between SH-SY5Y and LAN-5 cell lines, we chose miR-101, miR-34a, miR-424, miR-21, miR-504 and miR-92a which clearly represent the phenotypic differences between the two cell lines as concerns tumorigenicity and NB tumor progression, regulation of cell cycle, differentiation and targeting of MYCN. The distribution of the deltaCt values of all the miRNAs studied in 15 tumor tissue specimens resulted very heterogeneous as evidenced in the box plot shown (Fig. 3A). To obtain a more accurate analysis we divided the samples in two groups with high (≤ −1.00) and low (> −1.00) LMNA expression (threshold = LMNA deltaCt median value), including only those specimens whose difference between LMNA and MYCN expression was at least 4-fold (12 out of 15 tumor tissue specimens). Consistent with the changes observed in the two cell lines we found an increased expression of miR-101, miR-34a, miR-424, miR-21, and a decreased expression of miR-504 and miR-92a in the NB patient group with high LMNA expression compared with the specimen group with low LMNA expression (Fig. 3B).

### LMNA gene knock-down induces a stem-like phenotype in SH-SY5Y cells

**Figure 4:**
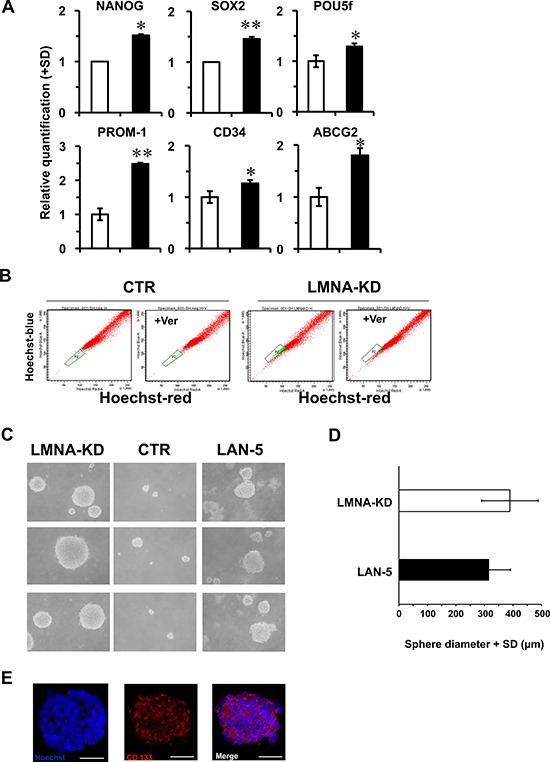
SH-SY5Y LMNA-KD cells acquire stemness characteristics **A.** qRT-PCR analysis of the indicated genes in control (CTR; white) and LMNA-KD (black) cells. The data are reported as the level of mRNA relative to the number of control cells and are the means+SD (*n* = 5). Statistical significance: *p ≤ 0.05, **p ≤ 0.01. **B.** Flow cytometric analysis of side population after staining with Hoechst 33342 in the absence or presence of verapamil to inhibit the functionality of the membrane ABCG2 efflux pump. **C.** Tumor sphere assay of LMNA-KD cells compared to control and LAN-5 cells; phase contrast micrographs of the secondary spheres, magnification 200X. **D.** Mean diameter (+SD) of the secondary spheres formed in LMNA-KD cells and compared with those obtained in LAN-5 cells (*p* = 0.18). The SH-SY5Y cells did not produce any measurable spheres but only aggregates. **E.** Representative confocal images of the secondary tumor spheres. CD133 immunostaining (red) and nuclear staining (blue). Scale bar: 50 μm.

We used as experimental model the SH-SY5Y cells in which we previously inhibited differentiation by LMNA knock-down (LMNA-KD) [5]. First, we analyzed the expression of some stemness-related markers such as NANOG, SOX2, POU5f, PROM-1, CD34 and ABCG2, by qPCR (Fig. 4A). All of these genes were overexpressed in LMNA-KD cells, indicating that lack of the LMNA gene could be associated with a more immature cell phenotype. The activity of ABCG2, which is a member of the ATP-binding cassette (ABC) membrane transporters, and present in many types of stem cells, was also verified evaluating by FACS analysis the exclusion of the fluorescent supravital dye Hoechst 33342 out of the cells. The cells able to pump outside the dye are concentrated in a tailing population exhibiting dim fluorescence relative to the majority of cells with bright fluorescence and they represent the so called “side population”, known to correlate with a stem-like phenotype. Indeed, LMNA-KD cells show the presence of a side population identified by a Hoechst 33342 low-staining fraction (approximately 5%; Fig. 4B). As control to confirm dye efflux and the probable presence of stem-like cells we inhibited the ABCG2 pump by verapamil. Consistently, verapamil treatment led to depletion of this side population in LMNA-KD cells. By contrast, control cells presented the same cytofluorimetric profile in both the presence and absence of verapamil.

We then investigated the ability to form spheres by LMNA-KD cells in the appropriate culture conditions. As a positive control, we used human NB LAN-5 cells, known to form spheres [3]. Similarly to LAN-5 cells, LMNA-KD cells formed secondary spheres in serum-free medium (Fig. 4C). Noteworthy, the mean diameter of the spheres measured in LMNA-KD cells was superimposable to that of the LAN-5 positive control cells (*p* = 0.18; Fig. 4D). By contrast, control cells did not show any significant capacity to form secondary spheres. We carried out immunofluorescence experiments on the spheres to detect the expression of known stemness markers, such as CD133, SSEA4 and SOX2. LMNA-KD secondary spheres were positive for all the markers considered (Fig. 4E and Supplementary Fig. 1).

### LMNA-KD sphere-derived adherent cells maintain stemness characteristics and acquire a more aggressive phenotype

**Figure 5:**
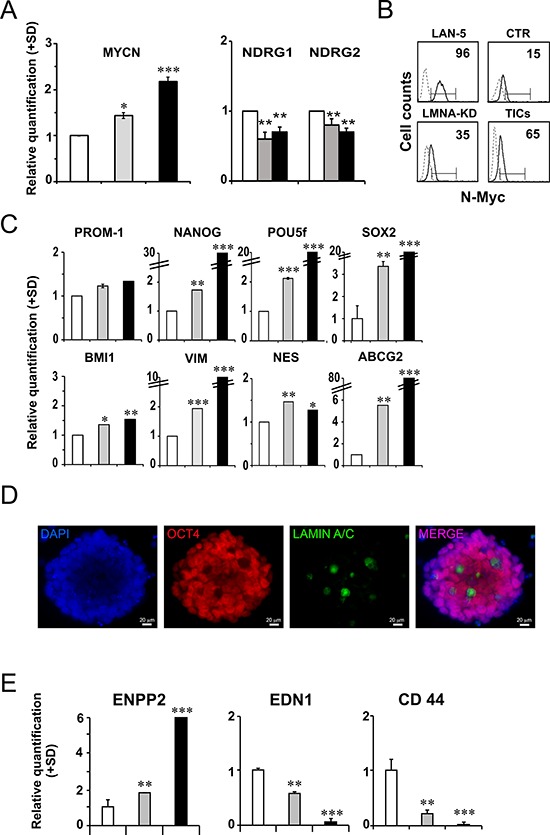
LMNA–KD sphere-derived adherent cells possess self-renewal and tumor progression characteristics **A.** RT-qPCR analysis of MYCN mRNA levels (right panel) and of NDRG1 and NDRG2 mRNA levels (left panel) in CTR cells (white), LMNA-KD cells (grey) and LMNA–KD sphere-derived adherent cells (black). The data are reported as the level of mRNA relative to CTR cells and are the means+SD (*n* = 5). Statistical significance: *p ≤ 0.05 **p ≤ 0.01, ***p ≤ 0.001. **B.** FACS analysis of N-Myc expression in control and LMNA-KD cells. LAN-5 cells were used as a positive control. The number reported in each histogram represents the percent positivity. **C.** RT-qPCR analysis of the indicated genes in CTR cells (white), LMNA-KD cells (grey) and the sphere-derived adherent cell line (black). The data are reported as the level of mRNA relative to LMNA-KD cells and are the means+SD (*n* = 5). Statistical significance: *p ≤ 0.05, **p ≤ 0.01, ***p ≤ 0.001. **D.** Representative immunofluorescence for Oct4 (red fluorescence) on mES culture, 4 days after exposure to growth medium lacking LIF interleukin, attesting the pluripotency of the cells. The absence of a signal for Oct4 and the presence of Lamin A/C (green fluorescence) demonstrate the luck of pluripotency and the beginning of the differentiation process. Nuclei were stained with DAPI (blue fluorescence). Scale bars: 20 μm. **E.** Relative expression of tumor aggressiveness-related genes in CTR cells (white), LMNA-KD cells (grey) and the sphere-derived adherent cell line (black). The data are reported as the level of mRNA relative to LMNA-KD cells and are the means+SD (*n* = 5). Statistical significance: **p ≤ 0.01, ***p ≤ 0.001.

Spheres formed from LMNA-KD cells were cultured in adherent conditions for several passages. Sphere-derived adherent cells grew as discrete groups of differently sized cells whereas the LMNA-KD cell monolayer showed a dispersed pattern, with growing cells being mainly isolated (Supplementary Fig. 2). We verified the expression of MYCN mRNA levels which significantly increased in LMNA-KD cells and in sphere-derived adherent cells (approximately 2-fold) compared with CTR cells thus confirming our hypothesis (Fig. 5A, left panel). The increased MYCN expression was associated with the down-regulation of the NDRG1 and NDRG2 genes in LMNA-KD cells and in sphere-derived adherent cells (Fig. 5A, right panel). An increase in the N-Myc protein accompanied the increase in MYCN gene expression in LMNA-KD cells and sphere-derived adherent cells, as evaluated by FACS analysis (approximately 40 and 65%, respectively; Fig. 5B).

We characterized the LMNA–KD sphere-derived adherent cells to evaluate the mRNA expression levels of self-renewal genes compared to those in the control cell line. The stemness marker genes maintained their expression or were further up-regulated in LMNA-KD sphere-derived adherent cells (Fig. 5C). In particular, we observed a strong up-regulation of the stemness network of the transcription factor genes NANOG, POU5f and SOX2 and of the drug resistance-related gene ABCG2. In addition, sphere-derived adherent cells maintained their self-renewal ability, as they were still able to form cell spheres in non-adherent culture conditions.

We used murine embryonic stem cells (mES) to study the expression of Lamin A/C protein in a pluripotent stem cell model. mES, cultured in stemness conditions, homogeneously showed the presence of OCT4 (data not shown). When Leukemia inhibitory factor (LIF, an interleukin 6 class cytokine that affects cell growth by inhibiting differentiation) was removed from the culture medium, allowing a generic differentiation, we detected the presence of Lamin A/C in those cells that did not express OCT4 anymore, suggesting that Lamin A/C could be an exclusive characteristic of the cells that begin the differentiation process (Fig. 5D).

Sphere-derived adherent cells appeared to acquire a more aggressive phenotype than LMNA-KD cells (Fig. 5E). The ENPP2 metastasis-associated gene showed a 4-fold increase with respect to LMNA-KD cells. In addition, the EDN1 and CD44 genes, known to be involved in cell adhesion functions, significantly decreased in the sphere-derived adherent cell line.

Mainly, these data strongly suggest that LMNA–KD sphere-derived adherent cells possess self-renewal and tumor progression characteristics that are peculiar of a putative population of tumor-initiating cells (TIC-like cells).

### The LMNA-KD sphere-derived adherent cell line is able to initiate tumors *in vivo*

**Figure 6:**
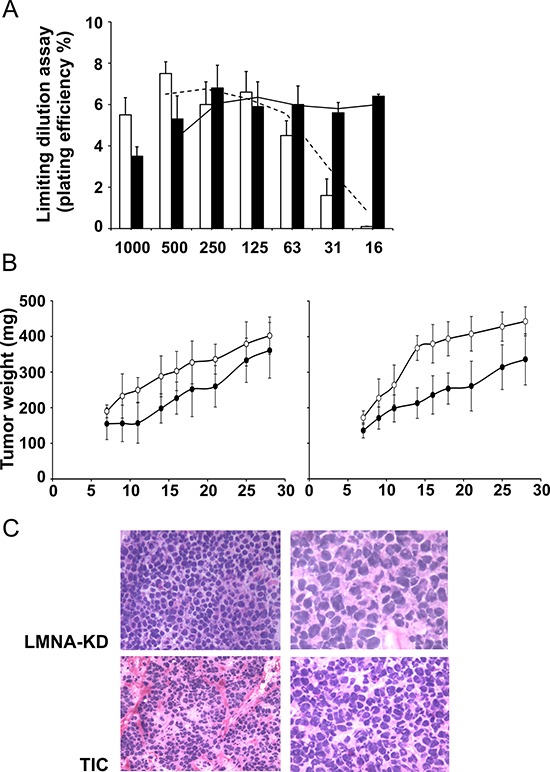
The LMNA-KD sphere-derived adherent cell line is able to initiate tumor growth *in vivo* **A.** *In vitro* plating efficiency (PE %) in LMNA-KD (white) and sphere-derived adherent (black) cells at different cell dilutions. **B.** LMNA-KD (•) or sphere-derived adherent (○) cells were injected subcutaneously into the flanks of immunosuppressed mice at 10^5^ (left panel) or 5 × 10^4^ (right panel) cells/mouse in 200 μl of Matrigel. When a tumor mass was evident, the tumor sizes were measured, and the tumor weight was calculated using the following formula: *a* × b^2^/2, where a and b are the long and short diameters of the tumor, respectively. The mean ± SD tumor weights are reported (*p* = 0.003, at day 14 and *p* = 0.01 at day 21 and 28 after tumor cell injection). **C.** Histopathological examination of tumor biopsies from LMNA-KD cells and TICs after 27 days of tumor growth. Images shown are at 20X (left panels) and 40X (right panels) magnification.

We performed a limiting dilution assay in LMNA-KD sphere-derived adherent cells (namely TIC-like cells in this paper) to determine the minimum cell dilution capable of forming colonies *in vitro*. While TIC-like cells maintained colony forming ability at very low dilution (down to 16 cells), LMNA-KD cells did not form colonies at such dilution (Fig. 6A).

On this basis, we compared the *in vivo* growth of the two cell lines using an *in vivo* limiting dilution assay. Immunosuppressed mice were injected subcutaneously with different number of LMNA-KD and TIC-like cells (from 5 × 10^4^ to 1 × 10^7^). No differences in terms of tumor appearance and tumor growth were evident injecting from 2.5 × 10^5^ to 1 × 10^7^ TIC-like and LMNA-KD cells (data not shown). While, at lower number, TIC-like cells exhibited a higher growth rate than LMNA-KD cells (Fig. 6B); this was evident when mice were injected with 10^5^ cells (left panel), but a more marked difference between TIC-like and LMNA-KD cells was found in terms of tumor mass when 5 × 10^4^ cells were injected per mouse (right panel). In fact, a significant increase in the weight of the TIC-like-derived tumors compared to the LMNA-KD-derived tumors (an almost doubling) was observed as the maximum effect (on day 14 after tumor cell injection) (*p* = 0.003). A significant difference between the two tumor growth rate was maintained also at day 21 and 28 after tumor cell injection (*p* = 0.01).

The histopathological pattern of these tumors, analyzed on day 27 of tumor growth, showed small cells with round to oval shapes, deeply stained nuclei and poorly defined cytoplasmic outlines. Unlike LMNA-KD-derived tumors, TIC-derived tumors were mainly microscopically characterized by a nodular pattern of growth. Small aggregates with mostly hyperchromatic nuclei were defined by the presence of fibrous septa. We also observed hypercellularity and overlapping nuclei occasionally exhibiting small nucleoli in TIC-derived tumors. These findings further demonstrate the more malignant phenotype of TIC tumors (Fig. 6C).

### MYCN expression is increased by miR-101 inhibitor in SH-SY5Y NB cells

**Figure 7:**
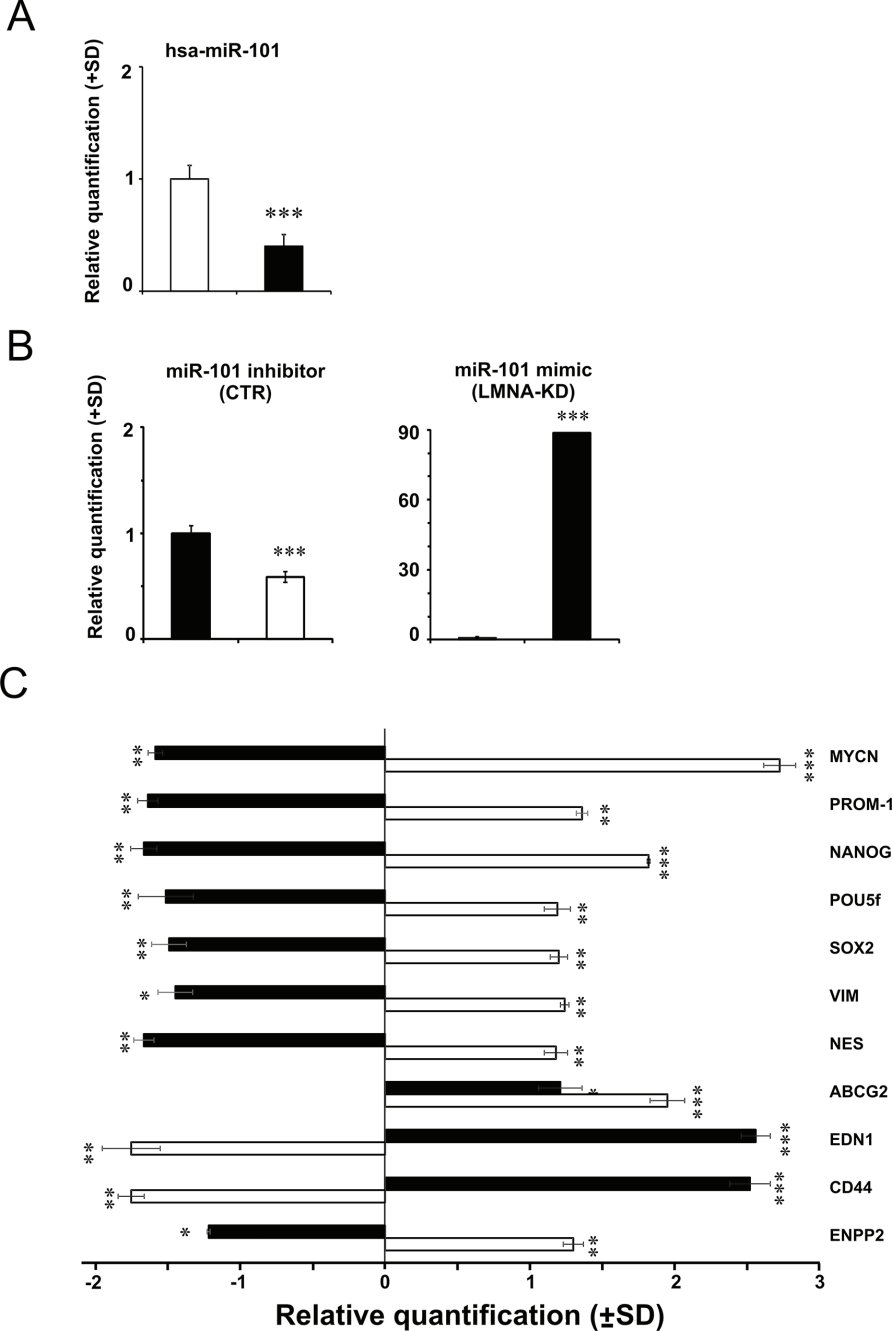
Modulation of hsa-miR-101 modifies the expression of MYCN gene and in turn of stemness and cancer-related genes **A.** TaqMan MicroRNA Assay with hsa-miR-101 in CTR cells (white) and LMNA-KD cells (black). The data are reported as the level of mature miRNA and mRNA relative to CTR cells and are the means+SD (*n* = 5). Statistical significance: *p ≤ 0.05, **p ≤ 0.01. **B.** TaqMan MicroRNA Assay, with the hsa-miR-101 levels in CTR cells transfected with the inhibitor (white, left panel) or in LMNA-KD cells transfected with the mimic (black, right panel). The data are reported as the level of mature miRNA compared to each relative negative control and are the means+SD (*n* = 5). Statistical significance: ***p ≤ 0.001. **C.** qRT-PCR analysis of the indicated genes in hsa-miR-101 mimic-transfected LMNA-KD cells (black) and hsa-miR-101 inhibitor-transfected CTR cells (white). The data are reported as the level of mRNA compared to each relative negative control and are the means+SD (*n* = 5). Statistical significance: *p ≤ 0.05, **p ≤ 0.01, ***p ≤ 0.001.

From a more accurate analysis of the table 1 we found that MYCN gene appeared as one of the miRNA target in the SH-SY5Y cells, whereas LMNA did not emerge as a miRNA-regulated gene in the LAN-5 cells, thus indicating that the absence of MYCN expression in SH-SY5Y cell line might be the results of miRNA regulation. Since miR-101 is a specific miRNA known to target also MYCN gene [19, 22], we inhibited miR-101 in SH-SY5Y control cells to modulate MYCN expression. As control, we overexpressed miR-101 in LMNA-KD cells in order to verify the effect on MYCN gene expression. As expected, the analysis of the intrinsic expression of miR-101 in both cell lines, revealed miR-101 less expressed in LMNA-KD cells by 50% compared to SH-SY5Y control cells (Fig. 7A).

Transfection of SH-SY5Y control cells with the miR-101 inhibitor decreased the expression levels of miR-101 by approximately 50% (Fig. 7B, left panel). Transfection of LMNA-KD cells with the miR-101 mimic resulted in approximately 90-fold increased expression of miR-101 (Fig. 7B, right panel). The analysis of the MYCN transcripts resulted significantly increased or decreased after transduction of miR-101 inhibitor or mimic, respectively (Fig. 7C). We then analyzed the mRNA levels of genes involved in some of the MYCN-regulated pathways such as stemness and aggressiveness. The expression of the same genes up-regulated in SH-SY5Y control cells after the inhibition of miR-101, decreased in LMNA-KD cells after the overexpression of miR-101. Consistently, after inhibiting miR-101, SH-SY5Y control cells showed down-regulation of the tumor suppressor EDN1 and CD44 genes, and increased expression of the tumor progression ENPP2 gene with respect to LMNA-KD cells transfected with the miR-101 mimic (Fig. 7C). We also verified the effect of the N-Myc transcription factor on the LMNA gene expression by analyzing the expression of LMNA gene in SH-SY5Y in which we overexpressed MYCN either by inhibiting miR-101 or by ectopically transducing a MYCN construct. In both cases we did not observe any increase in the expression of the LMNA gene and of the Lamin A/C protein (data not shown).

## DISCUSSION

This paper demonstrates that the down regulation of Lamin A/C expression in the SH-SY5Y NB cells allows the development of a tumor-initiating cell (TIC) population with self-renewal features and the ability to initiate tumors *in vivo*.

TICs have been identified in many tumor types as a small tumorigenic population of cells that are responsible for sustaining tumor growth and metastatic spread, thus representing the cause of cancer recurrence [23–25]. Their presence has been demonstrated in the bone marrow of patients with relapsed NB [2], and they have many stem cell characteristics, such as sphere-forming ability and self-renewal. However, sphere-forming ability is not a general characteristic of NB tumor-derived cell lines, as reported by Mahller and colleagues [3], who studied the capacity to give rise to spheres in eight NB cell lines, including SH-SY5Y. In accordance with these authors, we also found that NB SH-SY5Y cells were not able to form spheres. However, when we silenced the LMNA gene in these cells, they gave rise to spheres in serum-free culture conditions that were optimized for neural stem cell growth. The capacity to form spheres clearly demonstrates that this selected population possesses the self-renewal characteristics of a stem cell phenotype. Pluripotency is a unique state in which cells can self-renew indefinitely while maintaining the ability to differentiate. The mechanism that controls the transcription of core pluripotency factors has been extensively studied [26, 27]. In normal embryonic stem cells, the gene regulatory networks of the core transcription factors Oct4, Nanog and Sox2 are involved in pluripotency maintenance [27]. Consistently, we found that the three main genes belonging to the stem cell regulatory network (POU5f, which encodes Oct4, NANOG and SOX2) were all significantly overexpressed in LMNA-KD-derived tumor spheres and even more so when the sphere cell population was cultured as adherent cells.

In addition, the fact that these spheres are highly positive for the CD133, SOX2 and SSEA4 markers further supports the stem-like phenotype of LMNA-KD cells.

We also found in LMNA-KD-derived tumor spheres a marked increase in the expression of the ABC membrane transporter gene ABCG2. Increased expression of this gene is considered a major characteristic of the cancer stem cell population that is associated with different tumor types [28]. The increased expression of this drug resistance-related gene often correlates with the presence of a side population [29]. Indeed, we found the unambiguous presence of a side population completely abolished by the exposure to verapamil, a known inhibitor of the membrane drug efflux-pump, in LMNA-KD cells.

To further corroborate that this cell population with self-renewal characteristics was a TIC population, we studied the capacity of these cells to give rise to tumors. The analysis of tumor growth *in vivo* clearly demonstrated that the putative TICs were more markedly aggressive, as evidenced by their significant higher growth rate at low cell concentrations than the LMNA-KD whole cell population and by the more malignant phenotype of the tumor tissues. It is likely that the enhanced tumorigenicity of TICs may depend on their maintenance in supportive micro-environmental “niches”. In normal tissues a “niche” consists of a microenvironment formed by cellular and non-cellular components which together are able of housing and maintaining stem cell homeostasis. On the other hand, there is evidence that the normal cells that surround and infiltrate tumors secrete factors that promote the growth and progression of cancer. Vermeulen et al. [30] reported that the microenvironment was imperative for regulating functional activity associated to colon TICs. Similarly, Calabrese et al. [31] demonstrated that brain vascular niche is responsible for promoting self-renewal and accelerating tumor growth from brain TICs. As reported by others in different tumor types including neuroblastoma [2, 32] we also used Matrigel in our *in vivo* studies in order to provide to TICs a complete microenvironment and biological substrates to promote TIC xenograft tumor formation.

The aggressiveness of the TIC population was also evidenced by increase of ENPP2 and decrease of EDN1 and CD44 gene expression. We can therefore conclude that the LMNA-KD sphere-derived cells represent a TIC population.

The inverse relationship between LMNA and the NB marker MYCN found in the TICs indicate that these two genes could mutually regulate differentiation and stemness in NB. Indeed, TICs which express more MYCN than SH-SY5Y control cells and are silenced for LMNA gene, also show a significant increase of the NANOG, POU5f and SOX2 gene expression. This is supported by the data obtained from biopsies of human NB. Indeed, we found, for the first time in this paper, a defined inverse relationship between LMNA and MYCN gene expression in 23 human NB biopsies.

On the other hand, the miRNome analysis in two NB cellular model expressing LMNA or MYCN alternatively, evidenced changes of members of miRNA signatures which control cell proliferation and differentiation. In fact, in the SH-SY5Y LMNA expressing cells DAVID pathway analysis revealed that the miRNA target genes belonged mainly to pathways related to cell cycle regulation indicating that the expression of Lamin A/C is associated with a more mature phenotype. This confirm data of a previous paper from our laboratory where we demonstrated that LMNA knock-down inhibit differentiation processes. By the contrary, in the LAN-5 MYCN expressing cells the miRNA target genes identified belonged to pathways related to the inhibition of the developmental processes. In particular, some of these genes (SMAD2/3, FGF2) appear to be involved in stem cell differentiation processes [33, 34]. The very different number of miRNAs identified in each cluster comparing the two cell lines support our hypothesis. It is likely that miRNAs and their target genes in LAN-5 cells tend to display a low number of clusters as a consequence of reduced number of pathways to be regulated to maintain their stem-like phenotype. By contrast, SH-SY5Y cells need to control a higher number of pathways than LAN-5 cells, due to their more mature phenotype. On the other hand, it is important to highlight that the analysis has been performed considering uniquely validated target. The validation in NB human specimens of some of the relevant miRNAs differentially expressed in the two NB cell lines confirms in the tumor biopsies the expression profiles obtained in the two cell lines. This suggests that this group of miRNAs could be the basis for a further identification of a miRNA signature representative of tumor progression in relation to LMNA expression.

The MYCN crucial role in the control of tumor initiating cell development is further demonstrated by data obtained modulating MYCN expression through miR-mediated regulation. The choice to modulate MYCN by miR-101 mediated regulation is based on the analysis of the miRNA array performed in SH-SY5Y and LAN-5 cells (see supplementary table 1). We evidenced hsa-miR-101–3p and hsa-miR-34a-5p as specific miRNAs targeting MYCN gene [19]. To confirm that these miRNAs were expressed at higher levels in SH-SY5Y than in LAN-5 cells we performed a qRT-PCR which confirmed the array result (data not shown). In addition, only miR-101 expression was decreased in LMNA-KD compared to control cells; while the expression of miR-34a did not change (data not shown). This suggests that only the former miRNA could have a role in regulating MYCN mRNA after LMNA knock-down. In fact, up-regulating MYCN in SH-SY5Y control cells, they acquired a stem-like phenotype; while down-regulating MYCN in LMNA-KD cells, we were able to restore a more mature phenotype. On the other hand, overexpression of the MYCN gene correlates with increased stemness characteristics [35–37]. In addition, the role of the MYCN gene in promoting tumor invasion, particularly in NB cells, is well known [38–40]. However, LMNA gene expression did not change when we increased MYCN in SH-SY5Y cells, indicating that an inverse correlation between LMNA and MYCN does not exist. Moreover, an *in silico* analysis of the LMNA promoter did not evidenced the presence of the Myc family transcription factors peculiar consensus sequence.

Our data provide new mechanistic insight into the development of a stem-like NB TIC population. In a tumor-mass, TICs constitute a rare tumorigenic cell population responsible for sustaining tumor growth, metastases and relapse. Our data may provide opportunities to develop new personalized therapeutic strategies based on the molecular profile of the tumor.

## MATERIALS AND METHODS

### Human NB biopsy RNA extraction and real-time RT-PCR

Total RNA was extracted from twenty-three frozen biopsies of human newly diagnosed NBs obtained from Department of Pediatrics and Infantile Neuropsychiatry of Sapienza University, Rome using TRIzol reagent (Life Technologies) according to the manufacturer's instructions. RNA was reverse-transcribed, and real-time PCR performed as described below. The amplification efficiencies of the primers for MYCN and LMNA genes were tested and resulted identical (LMNA efficiency = 2.1; MYCN efficiency = 2.0). Institutional written informed consent was obtained from the patient's parents or legal guardians according to the local institutional guidelines.

### Cell line maintenance and sphere formation

The SH-SY5Y NB cell line was purchased from ATCC. In this study, we also used two SH-SY5Y-derived clones, LMNA-KD and control (CTR) cells that were grown as previously described [5]. The LAN-5 NB cell line (a gift from Dr. Doriana Fruci) was grown in RPMI-1640 medium (Gibco) supplemented with 10% FBS (Hyclone), 2 mML-glutamine and 1% penicillin/streptomycin in a fully humidified incubator containing 5% CO_2_ at 37°C. To obtain spheres, cells were plated in a serum-free media in 60-mm, low-attachment culture dishes at a density of 9.5 × 10^5^ cells/cm^2^ and cultured in Neurobasal Medium (Gibco) supplemented with 20 ng/ml Epidermal Growth Factor (EGF, Sigma), 40 ng/ml basic Fibroblast Growth Factor (bFGF, Sigma), 1% neuronal supplement N2 (Gibco) and B27 (Gibco), and 2 μg/ml heparin. Three days after seeding, the cells formed floating neurosphere-like structures that grew rapidly until day 7. Before these structures became necrotic, we harvested and suspended them in Accutase enzymatic solution (Gibco) for 5 min at 37°C and then mechanically dissociated into a single-cell suspension. The cells were re-seeded in the same conditions as above, and the secondary spheres were allowed to form. As positive and negative controls, we used NB LAN-5 and SH-SY5Y cell lines, respectively. The secondary spheres were measured using ImageJ software and the value of diameter expressed in μm. TICs were derived by harvesting the secondary spheres, treating them with Accutase enzymatic solution (Gibco) for 5 min at 37°C and then mechanically dissociating them into a single-cell suspension. These cells were seeded in adherent conditions in the presence of 1:1 mixture of Eagle's Minimum Essential Medium and F12 medium (Gibco) supplemented with 10% FBS (Hyclone), 2 mM L-glutamine, 0.5% non-essential amino acids, 0.5% sodium pyruvate and 1% penicillin and streptomycin. mES cells were cultured following the protocol provided by ATCC (ES-D3, CRL-1934 ATCC).

### Total RNA preparation

Total RNA was isolated using a Total RNA purification kit (Norgen Biotek). RNA quantity was determined by measuring absorbance at 260 nm using a NanoDrop UV-VIS spectrophotometer. The quality and integrity of each sample was confirmed using a BioAnalyzer 2100 (Agilent RNA 6000 Nanokit); samples with an RNA Integrity Number (RIN) index lower than 8.0 were discarded.

### PCR array

RNA was reverse transcribed using TaqMan MicroRNA Reverse Transcription kit (Applied Biosystems). cDNA was preamplified using TaqMan PreAmp Master Mix (Applied Biosystems). qRT-PCR was performed with an Applied Biosystem 7900HT thermal cycler using TaqMan human microRNA array (TaqMan Human microRNA Array A #4398977 and B v3.0 #442812; Applied Biosystems) according to manufacturer's instructions. The downstream analysis filtered out miRNAs not detected in both cell lines (293), whereas those specifically expressed either in LAN-5 or in SH-SY5Y were considered and reported as cell line-specific. Data were then normalized calculating the ΔCt value for single miRNA against the average of the specific controls for each card according to manufacturer's instructions. Differential expression analysis was performed according to ΔΔCt method and only RQ ≥ 2 fold-change were considered for further analysis. miRNAs clusters were generated through the DIANA web tool mirPath v2.0 using miRBase MIMAT IDs (Release 21) remapped to the newest human genome assembly (GRCh38) to avoid duplicate entries present in the previous release. Uniquely targets reported in TarBase database v7.0 were included in the clustering, predicted targets were not taken into account. False Discovery Rate (FDR) correction was applied to the original *p*-value and only clusters with corrected *p*-values < 0.05 were shown.

### Real-time RT-PCR analysis

RNA was reverse-transcribed and real time PCR analysis were performed as previously described [41]. The specific primers used (each at a concentration of 200 nM) are reported in Supplementary Table 2. Each experiment was performed in triplicate. The expression data were normalized using the Ct values of GAPDH and TBP.

### Side population

To analyze the side population phenotype, LMNA-KD cells were stained according to the protocol of Goodell et al. [42]. Briefly, 5.0 × 10^6^ cells/ml were suspended in pre-warmed DMEM. Hoechst 33342 (Sigma) was added at a final concentration of 5 μg/ml in the presence or absence of 50 μM verapamil (Sigma), and the cell samples were incubated for 90 min at 37°C with intermittent shaking. After incubation, the cells were washed with ice-cold PBS, resuspended in ice-cold PBS, and analyzed for Hoechst 33342 efflux with a FACS Aria II (Becton Dickinson). The Hoechst 33342 dye was excited at 375 nm, which is near-ultraviolet, and the resultant fluorescence was measured at two wavelengths, using 450/40 BP and 670 LP filters for the detection of Hoechst blue and red, respectively. All data were analyzed using the Diva 6.1 Software (Becton Dickinson).

### Tumor sphere CD133 immunofluorescence

Spheres were cultured as previously described, harvested and then allowed to spontaneously settle in a 50-mL polypropylene conical tube. Excess supernatant was removed. Spheres were dispensed in an 8-well μ-Slide (Ibidi) and allowed to settle, and Matrigel from a 5X Matrigel BME Coating Solution (Trevigen) was added to 0.1X. The cells were fixed in ice-cold methanol for 20 min and permeabilized in 0.5% Triton X-100 containing 0.3% serum for 10 min. Blocking was carried out in Tris-buffered saline (TBS) solution with serum (1:5) for 10 min. The primary antibody anti-CD133 (clone 293C3; Miltenyi Biotec) was diluted in the blocking solution with 0.5% Tween-20, and the spheres were incubated overnight. The cells were washed three times in TBS and incubated with goat anti-mouse Alexa 594 F(ab)2 (in TBS plus 0.5% Tween-20). After three washes in TBS, the nuclei were stained with Hoechst 33342 Images were acquired using a confocal laser scanning microscope (Leica Confocal Microsystem TCS SP5). The images were processed using Leica Application Suite 6000, the brightness and contrast of the acquired images were adjusted, and the figures were generated using Adobe Photoshop 7.0.

### Immunocytochemistry in murine ES

mES were fixed in 4% paraformaldehyde for 10 minutes and incubated overnight at 4°C with the following primary antibodies used at 1:100: mouse monoclonal anti-Oct4 (OCT4; BD) and goat anti-Lamin A/C (JOL2; Chemicon Int.). After three washes with PBS, the cells were incubated with a 1:100 dilution of fluorescent-conjugated secondary antibodies for 1 h at room temperature. Secondary antibodies were: donkey anti-mouse IgG Alexa-Fluor 555 (Jackson ImmunoResearch) and donkey anti-goat IgG Alexa-Fluor 488 (Jackson ImmunoResearch). Negative controls were tested by incubation of only the secondary antibody without a primary antibody incubation: all secondary antibodies were negative for non-specific staining. After three washes, the sections were mounted with VECTASHIELD (Vector Laboratories) plus DAPI.

### N-Myc FACS analysis

SH-SY5Y CTR, LMNA-KD cells and TICs were analyzed by indirect immunofluorescence. The cells were harvested, washed in cold 1X PBS and fixed (1 × 10^6^ cells/ml) in a solution containing cold acetone/methanol (1:4 v/v) in 50% 1X PBS. For each sample, 1 × 10^6^ cells were incubated with the human monoclonal antibody anti-N-Myc (Santa Cruz) in medium containing 10% FBS and 0.5% Tween 20 for 1 h at room temperature. After washing in PBS, the cells were incubated with FITC-conjugated goat anti-mouse IgG (BD, Pharmingen) in PBS for 50 min. After an additional wash in PBS, the samples were measured using a FACSCalibur cytofluorimeter (Becton Dickinson). Samples incubated with IgG isotype control antibody were used as negative controls, and NB LAN-5 cells were used as a positive control. The analysis was performed using the CellQuest software package. To determine the N-Myc immunofluorescence positivity we used the method of 2% of background.

### Colony-forming assay

The LMNA-KD and sphere-derived adherent cell line were seeded at decreasing clonal densities (range: 15–1,000 cells/dish) in 60-mm Petri dishes. Fifteen days after seeding, the cells were fixed for 10 min in ice-cold methanol. A solution of 0.5% crystal violet in 25% methanol was added to the monolayer for 30 min. The dishes were then washed with ddH_2_O, and the colonies (at least 50 cells) were counted. The results were expressed as plating efficiency (percentage of colonies formed from the number cells seeded).

### *In vivo* experiments

Six- to eight-week-old CD-1 male nude (nu/nu) mice weighing 22–24 g were purchased from Charles River Laboratories (Calco, Italy). The procedures involving mouse care were in compliance with the Regina Elena National Cancer Institute animal care guidelines and with international directives (directive 2010/63/EU of the European parliament and council; Guide for the Care and Use of Laboratory Animals, United States National Research Council, 2011). To evaluate the tumorigenic ability of LMNA-KD cells or sphere-derived adherent cells, the mice were injected subcutaneously into the left flank with various concentrations of cells (from 5 × 10^4^ to 1 × 10^7^ cells/mouse) in 200 μl of Matrigel (BD Biosciences-Discovery Labware). Each group included five mice. Tumor sizes were measured three times a week in two dimensions using a caliper, and tumor weight was calculated using the following formula: *a* × b^2^/2, where a and b are the long and short diameters of the tumor, respectively.

### Histopathological analysis

The engrafted tumors were fixed with 4% phosphate-buffered formalin, and paraffin-embedded sections were stained using hematoxylin and eosin (H&E). The sections were then subjected to morphological analysis.

### miRNA assays

Equal amounts of RNA were reverse transcribed with the TaqMan^®^ MicroRNA Reverse Transcription Kit (Applied Biosystems) according to the manufacturer's instructions, with a custom 1X RT primer pool (hsa-miR-101–3p ID 002253; U6 snRNA ID 001973; hsa-miR-424 ID 000604; hsa-miR-34a ID 000426; hsa-miR-21 ID 000397; hsa-miR-504 ID 002084; hsa-miR-92a ID 000431). Real time PCR analysis was performed with an Applied Biosystems 7900HT thermal cycler using 20X Individual TaqMan^®^ MicroRNA Assays.

### Hsa-miR-101 inhibitor and mimic

SH-SY5Y control and LMNA-KD cells were seeded at a density of 5 × 10^4^ cells/cm^2^. After 24 h, the cells were transfected overnight in the presence of 10% FBS with mirVana miRNA inhibitor, miRNA mimic or the respective negative controls. Lipofectamine RNAiMAX Reagent (Life Technologies) was used as the transfection reagent, according to the manufacturer's instructions. We transfected the has-miR-101 inhibitor (MH11414, Ambion) and the mimic (MC11414, Ambion) at a final concentration of approximately 50 nM. The mirVana miRNA inhibitor and mimic negative control were used at the same final concentration of 50 nM. Samples were harvested at 72 h to perform mRNA expression analysis.

### Statistical analysis

Student's *t* test (unpaired, two-tailed) was used for statistical comparison of different tumor weights and between two groups. If there were more than two groups, we used the one-way ANOVA test.

## SUPPLEMENTARY FIGURES AND TABLES


