# Supplementary material for: Down-regulation of the Lamin A/C in neuroblastoma triggers the expansion of tumor initiating cells
Source: Oncotarget. 2015 Sep 3;6(32):32821–40. doi: 10.18632/oncotarget.5104 (PMC4741732; doi:10.18632/oncotarget.5104)
Supplement: Supplementary file 1 [file oncotarget-06-32821-s001.pdf]

SUPPLEMENTARY FIGURES AND TABLES

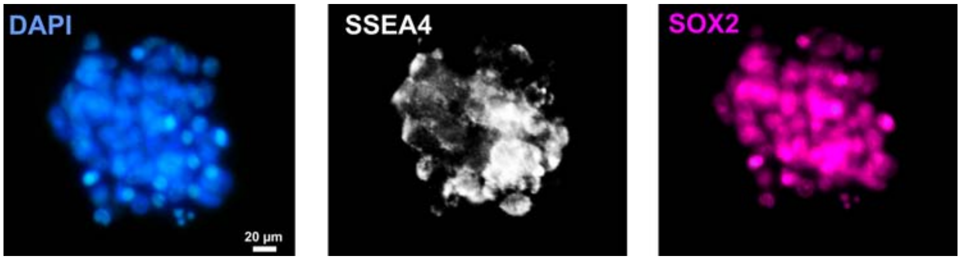

Supplementary Figure S1: Representative immunofluorescence for SSEA4 (Molecular Probes, cat. A24866) and SOX2 (Molecular Probes, cat. A24759) proteins on the secondary LMNA-KD derived tumor spheres. Both antibodies were used at 1:100 dilution. Nuclei were stained with DAPI. Scale bar: 20 μm.

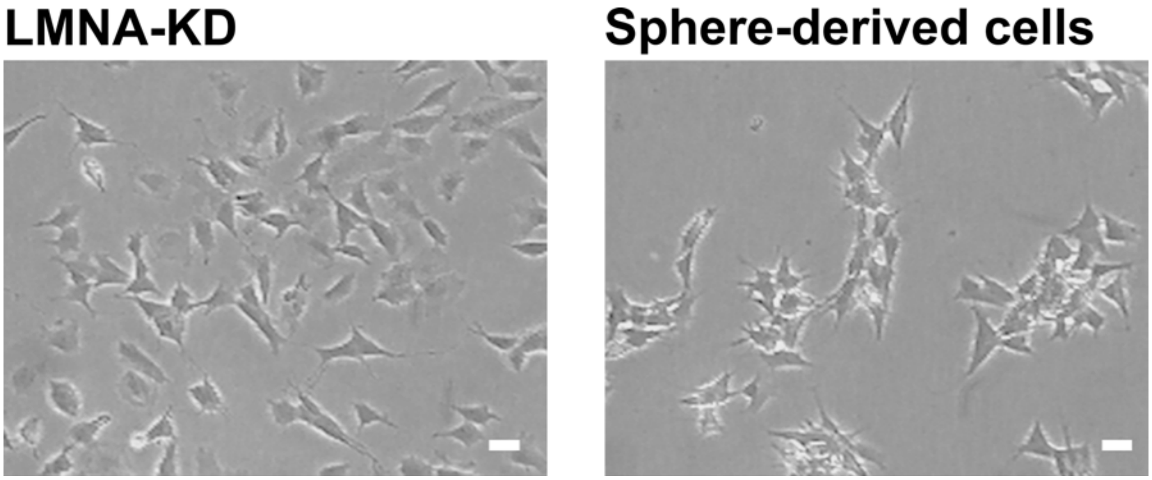

Supplementary Figure S2: Phase-contrast micrograph of LMNA-KD and sphere-derived adherent cells. Scale bar: 10 µm.

Supplementary Table S1: Functional analysis of differentially and specifically expressed miRNAs performed by DIANA tool showing statistically over-represented groups in SH-SY5Y and LAN-5 cells.

Increased* in SH-SY5Y

Term (KEGG_ Pathway)	Count	miRNA|TarBase				Count	*P* Value	Target Genes							
hsa04115: p53signaling pathway	19	miR-214–3p	miR-449a	miR-24–3p	miR-34a-5p	44	1.08E-29	CCNG1	ZMAT3	CCNB1	SFN	CDK4	BID	MDM2	RFWD2
		miR-222–3p	miR-21–5p	miR-374a-5p	miR-503			THBS1	CDK2	CCND2	DDB2	CDK1	GADD45A	CCND3	CDKN2A
		miR-424–15p	miR-193a-5p	miR-30d-5p	miR-101–3p			CDK6	CHEK1	TP53	APAF1	ATM	PMAIP1	PIDD1	CASP3
		miR-26b-5p	miR-26a-5p	miR-93–5p	miR-363–3p			CCND1	CCNE2	TP73	EI24	SESN1	CASP9	PPM1D	SESN2
		miR-28–5p	miR-28–5p	miR-10a-5p				MDM4	FAS	BBC3	CASP8	SERPINB5	TP53I3	CCNG2	CCNE1
								CDKN1A	RRM2	SESN3	PTEN				
hsa05200: Pathways in cancer	25	miR-214–3p	let-7c	miR-21–5p	miR-449a	86	5.47E-24	FOS	GSK3B	STAT3	E2F1	TGFBR1	ERBB2	COL4A1	CDK4
		miR-34a-5p	miR-222–3p	let-7f-5p	let-7d-5p			STAT5A	E2F2	NRAS	CRKL	BID	TCF4	PDGFA	APC
		miR-503	miR-424–5p	miR-223–3p	miR-27a-3p			RUNX1	WNT1	HDAC1	WNT5A	HSP90AA1	RALA	MDM2	CHUK
		miR-29b-3p	miR-363–3p	miR-146b-3p	miR-93–5p			BCL2	CDKN1B	BIRC5	PLD1	IGF1R	EGFR	TFG	CDKN2A
		miR-26a-5p	miR-361–5p	miR-25–3p	miR-361–5p			APPL1	KRAS	CDK6	VHL	TP53	IKBKB	TGFBR2	PTK2
		miR-28–5p	miR-28–5p	miR-24–3p	miR-769–5p			FZD4	MMP2	MAPK9	AR	JUN	CCND1	FGF1	SMAD4
		miR-374a-5p						CTNNB1	MSH6	CCNE2	AXIN2	MMP1	SKP2	MAPK1	COL4A2
								E2F3	MAPK8	MYC	MMP9	MSH2	DAPK1	FOXO1	PDGFB

(*Continued*)

Increased* in SH-SY5Y

Term (KEGG_ Pathway)	Count	miRNA|TarBase				Count	*P* Value	Target Genes							
								NFKBIA	KIT	PIK3R1	RB1	RAC1	BMP2	FGFR1	FAS
								TGFB2	EP300	BCL2L1	CCNE1	LEF1	CDKN1A	PTEN	MAP2K1
								Il6	BIRC3	RALB	STAT1	ITGA6	VEGFA		
hsa04110: Cell cycle	19	let-7c	miR-449a	miR-24–3p	miR-100–5p	51	6.56E-21	ESPL1	CDC6	GSK3B	CDKN2C	PCNA	E2F1	CCND3	CCNB1
		miR-34a-5p	miR-222–3p	miR-28–5p	let-7d-5p			SFN	CDK4	E2F2	CDC14A	CCNA2	CDC25C	CDC25A	HDAC1
		let-7f-5p	miR-503	miR-28–5p	miR-424–5p			MCM6	CCND2	MCM4	ORC4	CDKN1B	MCM5	MDM2	STAG2
		miR-363–3p	miR-27a-3p	miR-374a-5p	miR-26a-5p			WEE1	CDK1	GADD45A	CDKN2A	CDK6	MCM7	MCM3	CHEK1
		miR-93–5p	miR-25–3p	miR-769–5p				TP53	ATM	CDKN1C	CCND1	SMAD4	CCNE2	ANAPC13	E2F5
								SKP2	E2F3	MYC	RB1	CDC20	CDC23	PLK1	EP300
								CCNE1	CDC27	CDKN1A					

Increased* in LAN-5

Term (KEGG_ Pathway)	Count	miRNA|TarBase				Count	*P* Value	Target Genes							
hsa04110: Cell cycle	7	miR-376a-3p	miR-372	miR-193b-3p	miR-572	29	4.05E-32	ESPL1	CDC6	PCNA	E2F1	CCNB1	E2F2	CCNA2	MCM6
		miR-302d-3p	miR-942	miR-19a-3p				CDK1	CDK6	MCM7	CHEK1	CCND1	SMAD4	CCNE2	PKMYT1
								CDK2	CCND2	MCM4	MCM5	WEE1	ORC6	MCM3	CDC25A
								TTK	YWHAZ	CDC20	BUB1B	CDKN1A			

(*Continued*)

Increased* in LAN-5

Term (KEGG_ Pathway)	Count	miRNA|TarBase				Count	*P* Value	Target Genes							
hsa05200: Pathways in cancer	11	miR-182–5p	miR-378a-5p	miR-383	miR-942	31	1.12E-08	E2F1	RAC2	E2F2	RAD51	TCF7L1	ETS1	BCL2	KRAS
		miR-193b-3p	miR-572	miR-19a-3p	miR-491–5p			MSH6	CCNE2	MAPK8	NFKB2	HSP90AB1	CASP9	MAX	EP300
		miR-372	miR-200c-3p	miR-302d-3p				CDK6	MITF	CDH1	CCND1	SMAD4	BCL2L1	LEF1	FN1
								VEGFA	PTEN	FOXO1	TGFBR2	CDKN1A	SUFU		
hsa04115: p53 signaling pathway	8	miR-494	miR-130b-5p	miR-372	miR-19a-3p	15	1.35E-05	CCNB1	CDK2	CCND2	CDK1	CDK6	CHEK1	CCND1	CCNE2
		miR-193b-3p	miR-572	miR-942	miR-302d-3p			RCHY1	CASP9	SESN2	CDKN1A	RRM2	PTEN	PPM1D	
hsa04110: PI3K-Akt signaling pathway	7	miR-494	miR-182–5p	miR-383		13	8.07E-03	CDK2	CCND2	BCL2	CCND1	CCNE2	BCL2L1	FOXO3	CDKN1A
		miR-372	miR-19a-3p	miR-302d-3p				VEGFA	PTEN	SGK3	PRKCZ	BCL2L11			
		miR-491–5p													
hsa05202: Transcriptional misregulationin cancer	6	miR-370	miR-372	miR-572	miR-486–5p	4	4.32E-02	HMGA2	CD40	BCL2L1	CDKN1A				
		miR-491–5p	miR-942												

Specific of SH-SY5Y

Term (KEGG_ Pathway)	Count	miRNA|TarBase				Count	*P* Value	Target Genes							
hsa04110:Cell cycle	7	miR-221–3p	miR-302b-3p	miR-449b-5p	miR-34b-5p	11	9.08052E-10	CDK4	CCND2	CDKN1B	CDK6	CDKN1C	SMAD4	CCNE2	MYC

(*Continued*)

Specific of SH-SY5Y

Term (KEGG_ Pathway)	Count	miRNA|TarBase				Count	*P* Value	Target Genes							
		miR-675–5p	miR-34b-3p	miR-483–3p				RB1	CDC27	CDC25A					
hsa05200: Pathways in cancer	5	miR-199b-5p	miR-221–3p	miR-449b-5p	miR-34b-5p	12	5.24E-12	FOS	CDK4	BCL2	CDKN1B	CDK6	CCNE2	MYC	KIT
		miR-34b-3p						LAMC2	VEGFA	PTEN	DVL2				
hsa04115:p53 signaling pathway	8	miR-221–3p	miR-302b-3p	miR-449b-5p	miR-34b-5p	11	3.54E-11	CCNG1	CDK4	CCND2	CDK6	TP53	PMAIP1	CCNE2	SESN2
		miR-126–5p	miR-34b-3p	miR-483–3p	miR-1285–3p			BBC3	SESN3	PTEN					
hsa04110:PI3K-Akt signaling pathway	8	miR-181c-5p	miR-199b-5p	miR-221–3p	miR-302b-3p	20	1.52E-10	MYB	CDK4	PPP2CA	MCL1	CCND2	BCL2	CDKN1B	GNB1
		miR-449b-5p	miR-582–5p	miR-34b-5p	miR-34b-3p			PPP2R5C	KRAS	CDK6	DDIT4	CCNE2	MYC	KIT	HSP90B1
								FOXO3	LAMC2	VEGFA	PTEN				

Specific of LAN-5

Term (KEGG_ Pathway)	Count	miRNA|TarBase				Count	*P* Value	Target Genes							
hsa05200: Pathways in cancer	6	miR-96–5p	miR-155–5p	miR-504	miR-654–3p	40	2.58412 E-07	GSK3B	STAT3	NFKB1	SPI1	CDK4	SMAD2	E2F2	Spi1
		miR-630	miR-639					APC	WNT5A	CDK2	BAX	Csf1r	ETS1	SMAD3	BCL2
								EGFR	RHOA	CDKN2A	KRAS	Hif1a	MLH1	MITF	SMAD4
								CTNNB1	MSH6	CTNNA1	COL4A2	MSH2	RAC1	FGF2	FAS
								NKX3–1	CDKN1A	VEGFA	FOXO1	CSF1R	FGF7	JUP	MDM2

(*Continued*)

Specific of LAN-5

Term (KEGG_ Pathway)	Count	miRNA|TarBase				Count	*P* Value	Target Genes							
hsa04115: p53 signaling pathway	4	miR-96–5p	miR-504	miR-654–3p	miR-639	4	7.77E-04	CCNB1	BAX	FAS	CDKN1A	MDM2			
hsa04110: PI3K-Akt signaling pathway	3	miR-221–3p	miR-302b-3p	miR-449b-5p	miR-34b-5p	30	7.38E-03	GSK3B	NFKB1	CDK4	THBS1	PPP2CA	CDK2	PCK2	Csf1r
		miR-126–5p	miR-34b-3p	miR-483–3p	miR-1285–3p			ITGB4	BCL2	EGFR	KRAS	RHEB	ITGB5	COL4A2	PPP2R2A
								FLT1	PDK1	YWHAZ	RAC1	FGF2	EIF4E2	FOXO3	PKN2
								CDKN1A	SGK3	CSF1R	FGF7	GNB4	BCL2L11		
hsa04110: Cell cycle	4	miR-155–5p	miR-654–3p	miR-639	miR-376a-5p	15	2.16E-02	GSK3B	CDK4	SMAD2	E2F2	CDK2	SMAD3	STAG2	WEE1
								CDKN2A	SMAD4	TTK	YWHAZ	CDKN1A	PRKDC	PLK1	

*differentially expressed at least 2-fold comparing the two cell lines.

Supplementary Table S2: Primers sequences for SYBR green PCR assays.

Gene Symbol	Accession	F primer[a]	R primer[b]
ABCG2	Hs.480218	TGGCTTAGACTCAAGCACAGC	TCGTCCCTGCTTAGACATCC
BMI1	Hs.380403	TTCTTTGACCAGAACAGATTGG	GCATCACAGTCATTGCTGCT
CD34	Hs.374990	GCGCTTTGCTTGCTGAGT	GGGTAGCAGTACCGTTGTTGT
CD44	Hs.502328	GACACCATGGACAAGTTTTGG	CGGCAGGTTATATTCAAATCG
ENPP2	Hs.190977	GCACATCGAATTAAGAGAGCAG	GGGGGAGTCTGATAGCACTG
GAPDH	Hs.544577	AGCCACATCGCTCAGACA	GCCCAATACGACCAAATCC
MYCN	Hs.25960	CCACAAGGCCCTCAGTACC	TCCTCTTCATCATCTTCATCATCT
NANOG	Hs.635882	GATGCCTCACACGGAGACT	TTTGCGACACTCTTCTCTGC
NES	Hs.527971	TGCGGGCTACTGAAAAGTTC	TGTAGGCCCTGTTTCTCCTG
POU5f	Hs.249184	CTTTGAGGCTCTGCAGCTTAG	GGTTTCTGCTTTGCATATCTCC
PROM-1	Hs.614734	TCCACAGAAATTTACCTACATTGG	CAGCAGAGAGCAGATGACCA
SOX2	Hs.518438	TGCTGCCTCTTTAAGACTAGGAC	CCTGGGGCTCAAACTTCTCT
TBP	Hs.590872	GAACATCATGGATCAGAACAACA	ATAGGGATTCCGGGAGTCAT
VIM	Hs.455493	GTTTCCCCTAAACCGCTAGG	AGCGAGAGTGGCAGAGGA
